# GC content around splice sites affects splicing through pre-mRNA secondary structures

**DOI:** 10.1186/1471-2164-12-90

**Published:** 2011-01-31

**Authors:** Jing Zhang, CC Jay Kuo, Liang Chen

**Affiliations:** 1 Ming Hsieh Department of Electrical Engineering, University of Southern California, Los Angeles, California 90089, USA; 2 Molecular and Computational Biology, Department of Biological Sciences, University of Southern California, Los Angeles, California 90089, USA

## Abstract

**Background:**

Alternative splicing increases protein diversity by generating multiple transcript isoforms from a single gene through different combinations of exons or through different selections of splice sites. It has been reported that RNA secondary structures are involved in alternative splicing. Here we perform a genomic study of RNA secondary structures around splice sites in humans (*Homo sapiens*), mice (*Mus musculus*), fruit flies (*Drosophila melanogaster*), and nematodes (*Caenorhabditis elegans*) to further investigate this phenomenon.

**Results:**

We observe that GC content around splice sites is closely associated with the splice site usage in multiple species. RNA secondary structure is the possible explanation, because the structural stability difference among alternative splice sites, constitutive splice sites, and skipped splice sites can be explained by the GC content difference. Alternative splice sites tend to be GC-enriched and exhibit more stable RNA secondary structures in all of the considered species. In humans and mice, splice sites of first exons and long exons tend to be GC-enriched and hence form more stable structures, indicating the special role of RNA secondary structures in promoter proximal splicing events and the splicing of long exons. In addition, GC-enriched exon-intron junctions tend to be overrepresented in tissue-specific alternative splice sites, indicating the functional consequence of the GC effect. Compared with regions far from splice sites and decoy splice sites, real splice sites are GC-enriched. We also found that the GC-content effect is much stronger than the nucleotide-order effect to form stable secondary structures.

**Conclusion:**

All of these results indicate that GC content is related to splice site usage and it may mediate the splicing process through RNA secondary structures.

## Background

Pre-mRNA splicing in eukaryotes removes introns and joins exons together. It is catalyzed by the splicesome that is a large ribonucleoprotein complex with several hundred proteins and five small nuclear RNAs [1,2]. The recognition of splice sites requires multiple RNA binding proteins to bind to various splicing signals in pre-mRNAs. Genes can choose different sets of splice sites to produce multiple transcript isoforms, which further increases the complexity of splicing regulation. In eukaryotes, besides some short consensus sequence elements around the 5' splice site (5'ss), the 3' splice site (3'ss), the branch point, and the polypyrimidine tract, the splicing process needs other splicing regulatory elements (SREs) such as splicing enhancers or silencers [3-6]. In addition, pre-mRNA secondary structures also play an important role in splicing regulation [7].

It has been reported that local RNA secondary structures affect splice site selection through experimental observations from individual genes [8-12]. With the growing amount of genomic data and tools available, more genome-wide studies were carried out to support the hypothesis that pre-mRNA secondary structures are involved in the splicing process. For example, Patterson *et al*. [13] reported that the splice site prediction can be improved by adding the localized pre-mRNA secondary structure information to the conventional sequence-based approaches. Hiller *et al*. [14] found that some experimentally verified splicing enhancers and silencers near splice sites are significantly enriched in the single-stranded regions of the local secondary structures. Conserved secondary structures in *Drosophila *genomes were identified and they may modulate splicing regulation through long distance interactions [15]. Shepard *et al*. [16] discovered that stable and conserved pre-mRNA secondary structures around splice sites may promote alternative splicing to a large extent. All of these results indicate that the secondary structure of pre-mRNA is part of the mRNA splicing code [6,17,18].

In this work, we first confirmed that for internal exons, structures around alternative splice sites are significantly more stable than those around constitutive and skipped splice sites in multiple species. More importantly, we found that these splice sites have distinct GC content. The GC content differences can remarkably explain these stability differences because GC content is positively associated with structural stability and sites with thermodynamic advantages tend to be GC enriched. We also found that splice site of the first exon in humans and mice tends to be more stable, no matter whether it is an alternative splice site or a constitutive splice site, because the promoter regions are generally GC enriched [19]. In addition, splice sites of long exons tend to be GC-enriched and hence more stable in the structural level. Tissue-specific alternative splice sites in humans are also GC-enriched, which indicates the functional consequence of the GC effect. We further show that regions around splice sites are GC enriched as compared to regions far away or decoy splice site regions, which suggests a selection pressure on splice site regions to form stable secondary structures. By contrast, the nucleotide-order effect to the structural stability around splice sites is insignificant. All these support that GC may be an important factor in splicing through forming stable secondary structures.

## Results

### Difference of pre-mRNA secondary structures between alternative, constitutive, and skipped splice sites

**Figure 1:**
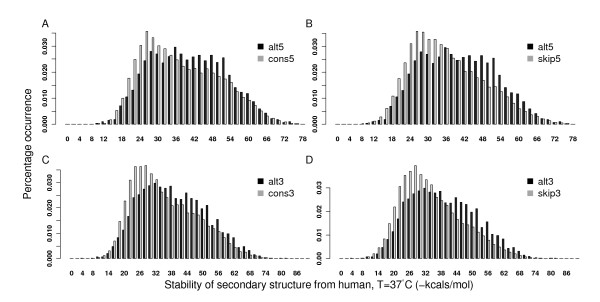
**Comparison of stability distribution of alternative splice sites and constitutive or skipped splice sites in humans**. A and B are for the 5'ss comparison. C and D are for the 3' ss comparison. "alt" means alternative splice sites, "cons" means constitutive splice sites, and "skip" means skipped splice sites. Alternative splices sites tended to have more stable structures.

It has been reported that stable RNA secondary structures are associated with alternative splicing events [16]. We first examined the stability of RNA secondary structures near exon-intron junctions in humans. Specifically, we assembled alternative splice sites from internal exons with multiple splice sites, constitutive splice sites from internal constitutive exons, and skipped splice sites from cassette exons (see methods). Since pre-mRNA sequences favor local structures rather than global ones in *vivo *[20], 70 nucleotides were added up- and down-stream of each splice site to predict the secondary structure by the free energy minimization program RNAfold [21,22]. The structural stability distribution in humans is plotted in Figure 1. At the donor sites (5'ss), the average minimum free energy of alternative splice sites was -41.28 kcals/mol, significantly lower than those of constitutive and skipped splice sites: -38.43 and -37.20 kcals/mol respectively (Wilcoxon tests, *P *< 2.2 × 10^-16^, Figures 1A and 1B). Similarly, at the acceptor sites (3'ss), the average free energy around alternative splice sites was -40.03 kcals/mol, compared with -36.18 and -35.28 kcals/mol for constitutive and skipped splice sites (Wilcoxon tests, *P *< 2.2 × 10^-16^, Figures 1C and 1D). The comparison demonstrates that for internal exons, alternative splice sites favor more stable secondary structures than constitutive and skipped splice sites, which is consistent with the results in [16]. In order to test whether this thermodynamic advantage of alternative splice sites might have existed from ancient times, we generalized our structure stability comparison in several other species as well. For all of the species that we have tested (nematodes, fruit flies, and mice), a significant enrichment of stable structures was observed in alternative splice sites except that the difference between mouse alternative donor sites and constitutive donor sites was small (Additional files 1, 2, and 3). Due to the incompleteness of transcript annotations, it was difficult to distinguish alternative splice sites from the constitutive and skipped ones with high confidence in many other species.

### Difference of GC content between alternative, constitutive, and skipped splice sites and its association with the stability of pre-mRNA secondary structure

**Figure 2:**
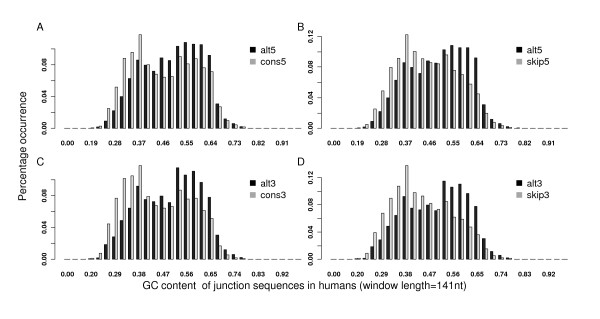
**Distributions of the GC content of different splice sites in humans**. Compared with constitutive and skipped splice sites, alternative splice sites were significantly enriched with GC (the p-values based on the Wilcoxon tests were all < 2.2 × 10^-16^).

In the exploration of other differences between different splice sites, we found that alternative splice sites tend to be GC enriched. For example, at the donor sites of the human genome, the average GC percentage was 0.52 for alternative 5'ss, which was significantly higher than 0.48 and 0.47 for constitutive and skipped splice sites (Wilcoxon tests, *P *< 2.2 × 10^-16^, Figures 2A and 2B). At the acceptor sites, the GC content of alternative 3'ss was also significantly higher than that of constitutive or skipped splice sites: 0.52, 0.47, and 0.46 respectively (Wilcoxon tests, *P *< 2.2 × 10^-16^, Figures 2C and 2D). Results were similar when a 61-nt instead of the 141-nt sequence window was used for each splice site (Additional file 4).

**Figure 3:**
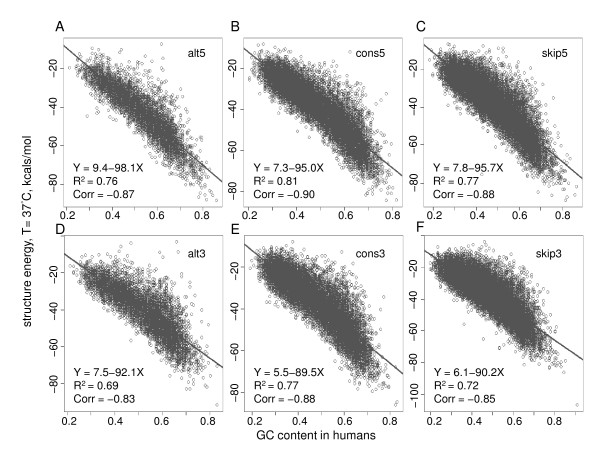
**GC content negatively correlates with energy in humans**. A-C are for alternative, constitutive, and skipped 5'ss. D-F are for alternative, constitutive, and skipped 3'ss. The GC content in all splice sites in humans was negatively correlated with the predicted structural energy. The fitted regression lines and the R^2 ^values are listed to show the goodness of fit.

**Figure 4:**
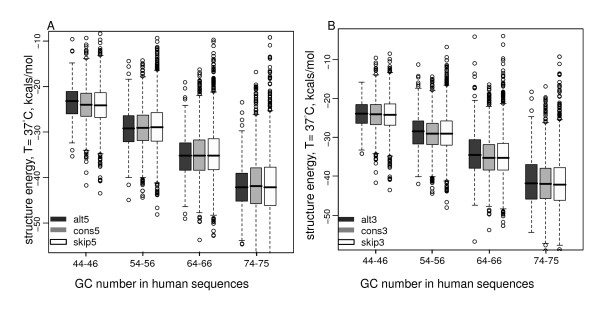
**GC content difference explains structural stability difference**. Boxplots of the energy are plotted (dots represent outliers). Alternative, constitutive, and skipped splice sites in humans had similar stability given the similar GC number (Wilcoxon tests for splice site energy comparison within each GC group, *P *> 0.05).

We then studied the relationship between the structural difference and the GC difference between alternative splice sites and constitutive or skipped splice sites. Linear regression was performed to reveal that the GC content difference can explain the stability variations among these splice sites remarkably. Specifically, the GC content was negatively correlated with the predicted minimum free energy of the sequence (Pearson's correlations in humans: -0.90~-0.83, *P *< 2.2 × 10^-16^). In other words, GC content was positively correlated with stability. Such significant correlations were observed for each splice site category, and for both the donor and acceptor sites (Figure 3). We further found that the overall GC content, no matter if it is intronic or exonic, contributes to the correlation with the energy significantly (Additional file 5). The fitted regression lines between the GC content and the energy were similar in different splice site categories. It indicates that alternative, constitutive, and skipped splices sites have similar structural stability given the same GC content. Similar results were observed in nematodes, fruit flies, and mice (Additional files 6, 7, and 8). The regression lines of nematodes and fruit flies were slightly different from those of humans and mice, possibly due to the biological differences between these organisms. In the RNAfold program, we set different temperature parameters to reflect their different body temperatures. We repeated the free energy analysis in nematodes and fruit flies by setting the same temperature parameter as that in humans and mice, and still observed regression lines different from those in humans and mice, indicating that such differences were not simply due to the different temperature settings (Additional files 9and 10). To further demonstrate that the GC content difference can explain the stability variations between alternative and constitutive or skipped splice sites, we compared alternative splice sites with constitutive and skipped splice sites with similar GC content in humans (Figure 4). No significant energy difference was observed among these splice sites given the same GC content (Wilcoxon tests, *P *> 0.05), indicating that the GC percentage of the junction sequence is the major factor to explain the distinct potentials to form stable structures among alternative, constitutive, and skipped splice sites.

Since structural stability was significantly associated with the GC percentage in junction sequences, it is possible that long-range GC enrichment patterns, instead of the local GC variation, would result in the genome-wide thermodynamic advantages in alternatively spliced sites. For example, if alternative splice sites were more frequently selected in high GC isochors, the junction sequences therein would be significantly biased to more stable base pairings compared with the constitutive and skipped splice sites. To clarify these two factors, pairwise free energy comparisons were performed with strict distance control to ensure similar GC background but allowing local GC variations. Across the human genome, we only selected the alternative splice sites with at least one constitutive splice site within a distance of 3000 bp. The alternative splice sites and the nearby constitutive splice sites shared similar GC background, but had local variations. Pairwise energy comparison of these two groups still suggested slight yet significant enrichment of more stable structures in alternatively spliced sites (-45.54 vs. -44.71 kcals/mol, paired Wilcoxon test *P *= 0.001 for the donor sites, -43.95 vs. -43.46 kcals/mol, *P *= 0.03 for the acceptor sites). Similar results were also observed in the comparison between alternative and skipped splice sites (data not shown), further confirming the contribution of local GC variations to stabilize mRNA secondary structures.

### Splice sites of the first exons or long exons are GC enriched and hence more stable in humans and mice

It is well known that human promoter regions are enriched in GC [19]. We suspect that splice sites near transcript start sites may form more stable structures compared with those in internal regions. To test our hypothesis, a list of constitutively and alternatively spliced donor sites of the first exons was generated and structures were predicted by the RNAfold program (see methods). Note that for studies in other places of this paper, only splice sites of internal exons were considered. As expected, both alternative (-52.87 vs. -41.28 kcals/mol) and constitutive splice sites (-55.56 vs. -38.43 kcals/mol) near the transcription start sites preferentially formed more stable structures than the middle ones (Wilcoxon tests, *P *< 2.2 × 10^-16^). However, in contrast with the splice sites of the middle exons, for the first exons, alternative splice sites displayed less stable structures than constitutive splice sites (-52.87 vs. -55.56 kcals/mol, Wilcoxon test, *P *= 4.9 × 10^-4^). As expected, the GC content of these alternative splices was lower than that of the constitutive splice sites (0.62 vs. 0.64, Wilcoxon test, *P *= 7.0 × 10^-6^). The results indicate that the intervention of RNA secondary structures in splicing may vary upon regions and it depends on GC content.

Shepard *et al*. [16] found that long exons tended to have more stable structures around splice sites. Similarly, we observed a significant bias in long exons (length > 200 bp) toward more stable structures in humans (Wilcoxon tests, *P *< 2.2 × 10^-16 ^for the donor sites and *P *= 5.3 × 10^-8 ^for acceptor sites). More importantly, this difference can also be explained by the GC difference. Specifically, the GC content of the splice sites around long exons was higher compared with that around short exons (length ≤ 200 bp) (Wilcoxon tests, *P *< 2.2 × 10^-16 ^for both the donor and acceptor sites). Hence, these results suggest that pre-mRNA secondary structures may play different roles in the splicing of exons with different lengths, depending on the GC content.

Similar results for the first exons and long exons were obtained for mice. However, in nematodes or fruit flies, since promoter regions were not GC-enriched [23], the splice sites near the promoter regions did not exhibit more stable structures. Besides, no obvious bias toward more stable structures has been observed in long exons of nematodes or flies.

### Tissue-specific alternative splice sites are GC enriched

**Figure 5:**
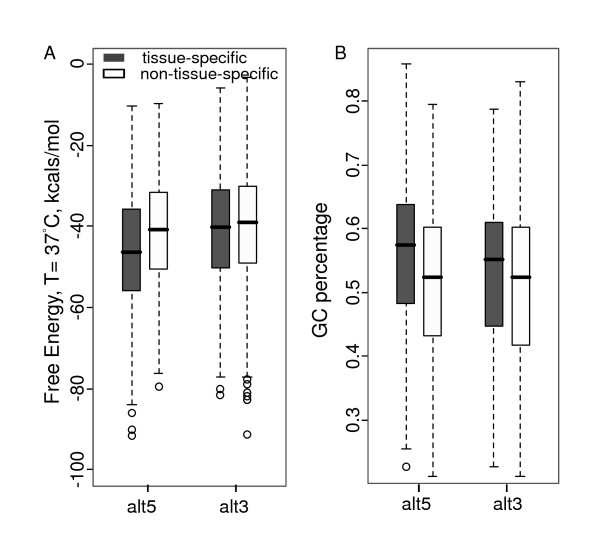
**Free energy and GC content comparison between tissue-specific and non-tissue-specific alternative donor and acceptor sites in humans with boxplots**. A is for the energy comparison between tissue-specific and non-tissue-specific splice sites in humans. The average free energy at the donor sites was -46.25 kcals/mol for tissue-specific and -41.20 kcals/mol for non-tissue-specific donor sites (Wilcoxon test, *P *< 2.2 × 10^-16^). At the acceptor site, the average energy was -40.65 vs. -39.98 kcals/mol (Wilcoxon test, *P *= 0.04). B is for the GC content comparison. The average GC content for the tissue specific donor sites was 0.56, and decreased to 0.51 for non-tissue-specific ones (Wilcoxon test, *P *< 2.2 × 10^-16^). Similar results were obtained at the acceptor sites (0.53 vs 0.51, Wilcoxon test, *P *< 4.9 × 10^-5^).

To investigate the consequence of the GC effect, we focused on tissue-specific alternative splicing events that are more likely to be functional. Similar to the criteria in [24], splicing events with a proportionality change of at least 10 percent and a corresponding P-value less than 0.3 in any of the 48 human tissues were considered as tissue-specific events. A total of 1,640 alternative donor sites and 1,342 alternative acceptor sites were claimed as "tissue-specific alternative splice sites" (see methods). We observed a significant bias of these functional splice sites to be GC-enriched, and thus to form more stable structures than other splice sites (Figure 5). For example, at the donor sites, the average energy for the tissue-specific alternative splice sites was -46.25 kcals/mol. It increased to -41.20 kcals/mol for the non-tissue-specific alternative splice sites (Wilcoxon test, *P *< 2.2 × 10^-16^). This stability difference can also be explained by the GC content difference (0.56 vs. 0.52, Wilcoxon test, *P *< 2.2 × 10^-16^). Similar results were also observed at the acceptor site.

### Selection on GC content around splice sites

**Figure 6:**
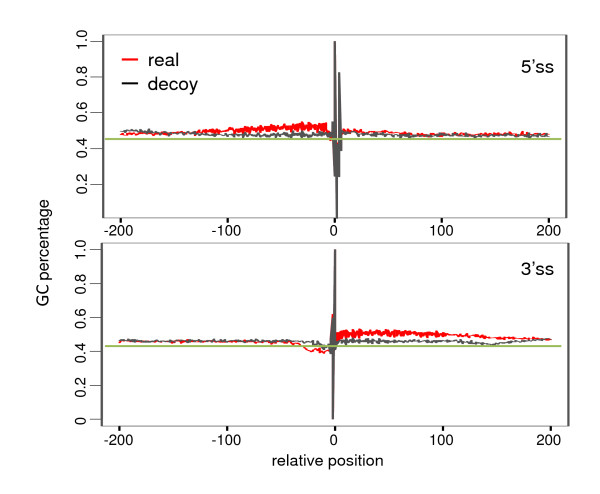
**Comparison of GC percentage around real (red lines) and decoy (black) splice sites in humans**. In general, the sequences around the real splice sites in humans were more GC enriched compared to the decoy ones (0.50 vs. 0.48 at the donor sites, 0.47 vs. 0.46 at the acceptor sites, Wilcoxon tests, *P *< 2.2 × 10^-16^). In addition, the exonic regions near the real splice sites were more enriched in GC compared to regions far away from the junctions, while no such enrichment has been observed near the decoy splice sites.

We have shown that GC content was strongly correlated with structural stability. We next investigated whether GC is specifically enriched around splice sites so that the formed stable structures can be involved in the splicing process. All the nucleotide sequences were aligned according to the exon-intron junction, and the average GC percentage across multiple sequences was calculated for each relative position. At the donor sites, in both exonic and intronic regions, the GC content around the splice sites was higher than that far away from the splice sites (red lines in Figure 6). At the acceptor sites, the exonic regions around the junctions were also enriched with GC. However, no significant GC enrichment was observed for the flanking introns around the acceptor sites, possibly due to the polypyrimidine track around these regions. In addition, we used the decoy splice sites [25] as controls (black lines in Figure 6). For these decoy splice sites, the GC content was almost uniformly distributed and there was no enrichment around the decoy splice sites. For each real splice site, we chose the closest decoy splice site as a control. Therefore, the real splice sites and the decoy splice sites shared the same GC background, but had local GC variation. In general, the real splice sites had higher GC content than the nearby decoy splices sites (paired Wilcoxon tests, *P *< 2.2 × 10^-16^), hence the real splice sites formed more stable structures (paired Wilcoxon tests, *P *= 1.38 × 10^-15 ^at donor sites, and *P *= 1.52 × 10^-8 ^at the acceptor sites). The difference between real splice sites and decoy splice sites could also be related to the higher GC content in the whole real exon region besides the splice site region. Then we calculated the GC content for the 50-bp exonic region near the real splice site, and then normalized it by the average GC percentage of the exon (i.e. the region from the junction site to 100 bp in the exonic direction). The GC content around the decoy splice sites was also normalized by their nearby 100-bp regions. After the normalization, the higher GC content due to exons was removed and we still observed GC enrichment in real splice sites compared with decoy splice sites (paired Wilcoxon tests, P = 2.25 × 10-4 for the acceptor sites, and *P *< 2.2 × 10^-16 ^for the donor sites). Thus, GC participates in both the exon formation and the splicing process. In addition, we found that the stability difference between real splice sites and decoy sites was larger for alternative splice sites than that for constitutive or skipped splice sites (1.83 vs. 1.38 or 0.68 kcals/mol at the donor sites, 1.00 vs. 0.71 or 0.44 kcals/mol at the acceptor sites). All these results indicate that real splice sites tends to be GC enriched, especially around alternative splice sites.

### GC effect is more dominant than the nucleotide-order effect

**Figure 7:**
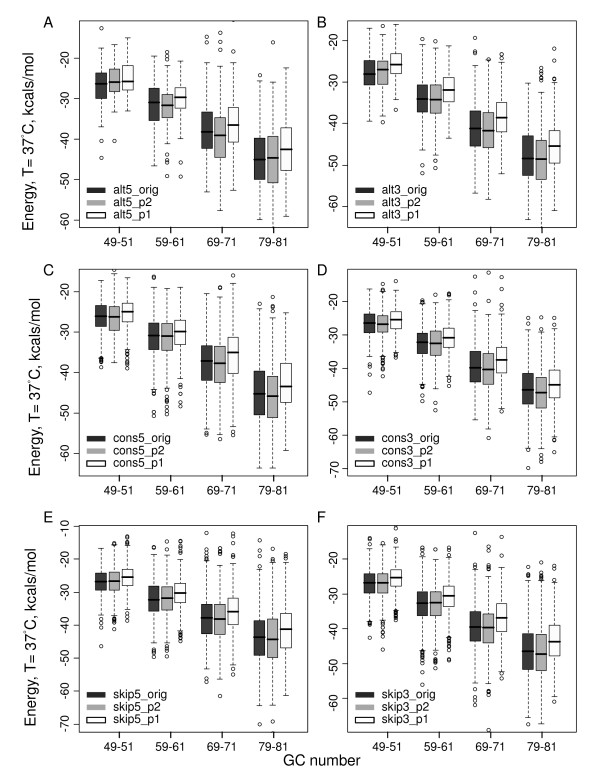
**Stability of native and permuted sequences in humans with boxplots**. A and B are for alternative splice sites. C and D are for constitutive splice sites. E and F are for skipped splice sites. The permuted sequences ("_p1",white) that kept the first order nucleotide frequencies always had less stable structures than the native sequences ("_orig", black). The difference was significant but relatively small compared with the difference between different GC-number groups. However, when the dinucleotide frequencies were fixed ("_p2", grey), the permuted sequences exhibited comparable structural stability, indicating insignificant contribution from the nucleotide order.

It has been reported that native mRNA sequences usually demonstrate lower minimum free energies as compared to permuted control sequences [26,27]. In this work, we focused on regions near the splice sites and compared the GC effect with the nucleotide-order effect. It is also known that the dinucleotide frequency affects the predicted free energy significantly due to the algorithm used in the RNAfold program, and the difference between native sequences and permuted sequences diminished if the dinucleotide frequency was fixed [27]. Therefore, in our analysis, both permutations keeping the first order nucleotide frequencies and permutations keeping the second order nucleotide frequencies were used. As shown in Figure 7, the GC percentage was correlated with the structure stability for all native and permuted sequences. Compared with the first order permuted sequences ("_p1", white boxes), the native sequences ("_orig", black boxes) showed more stable structures (paired Wilcoxon tests, *P *< 2.2 × 10^-16^). Nevertheless, when the dinucluetide frequencies were fixed, the energies of the permuted controls were similar to those of the native sequences, suggesting insignificant nucleotide-order effect with fixed dinucleotides frequencies. In addition, the difference between native and the first order permuted sequences increased with the GC content. For example, the mean energy change was -0.89 for the GC number around 50 and it was -2.19 for the GC number around 90 at the constitutive donor sites. Furthermore, the GC effect was more dominant than the first-order-nucleotide effect. In Figure 7, there are four GC number groups and the average GC number difference between adjacent groups is 10. The native sequences always showed less stable structures than the first order permuted sequences in adjacent groups that contain larger GC content (Wilcoxon tests, *P *< 2.2 × 10^-16^). For example, the native sequences with the GC number 49-51 were less stable than the first order permuted sequences with the GC number 59-61. Thus, the difference caused by the nucleotide-order effect (from the comparison of the "_orig" and "_p1") was smaller than that caused by the GC difference (i.e. the GC number difference of 10 or the GC percentage difference of 3.5%).

## Discussion

In this paper, we studied GC content around splice sites in terms of its effect on the splice site usage and the stability of pre-mRNA secondary structures in multiple species. For middle exons, alternative splice sites were more enriched with GC than constitutive and skipped splice sites, and hence exhibited stronger potential to form stable secondary structures (as shown in Figures 1 and 2). More importantly, we showed that the GC content was the major factor to account for the structural stability discrepancy. Given the same GC content, the predicted free energy is similar no matter whether it is alternative splice sites or other splice sites (Figure 4). We also notice that although the structural topology predicted from RNAfold has already been proved to demonstrate significantly high correlations with the experimental results in yeast [28], it is still possible that the estimated secondary structure may be different from that *in vivo *for the species we tested. In addition, it is still unclear whether RNA tertiary structure is also a common mechanism to regulate splicing [17]. Advanced experimental technologies are in need to better predict RNA structures.

Several possible regulation mechanisms can be proposed for our genome-wide structural stability observations. First, mRNA secondary structures may mediate the splicing process via affecting the motif recognition rate to facilitate or prevent the binding of splicing regulators. It has been reported that splicing regulators have different RNA structural topology preferences [29]. The stable stem regions (tend to be GC-enriched) in alternative splice sites might mask some key motifs and thus repress the site recognition. Second, long exons exhibit pronounced GC enrichment and hence more stable structures, indicating that RNA secondary structures may be actively involved in the splicing event via long distance mediations to bring the distal signals together [30,31]. Third, the GC-enriched sequences around the first donor sites indicate that the role of stable RNA secondary structures in splicing may vary across regions. It has been reported that splicing near the promoter region enhances transcription [32]. Our discovered stable secondary structures around the promoter regions may contribute to the interaction between splicing and transcription via serving as stable binding platforms of the transcription/splicing regulators. Furthermore, increasing evidence shows that in the beginning of the transcription process, the polymerase II may fall into some paused status, or even go forward and backward from time to time. Theoretical computations demonstrate that the stable hairpins in the nascent RNA is beneficial for the backtracking of polymerase II [33], which explain our discovery about the more stable structures of the first donor sites.

It is well known that the structure prediction software, such as RNAfold and Mfold, executes the pre-mRNA secondary structure calculation under simplified conditions, probably resulting in inappropriate minimum free energy predictions. Besides, the folding of nascent mRNA sequences may change frequently in different biological environments [34]. We therefore performed studies on functional alternative splice sites (i.e., tissue-specific alternative splice sites). These sites also tended to be GC-enriched, further suggesting the functional consequence of GC content in splicing.

Sequences around splice sites were more GC enriched compared to either the positions far away from the splice sites or nearby decoy splice sites, indicating the selection pressure on splice site regions to form stable structures. We also investigated whether additional factors exist to affect the structural stability. Permutation analysis reveals only limited nucleotide-order effect in the native sequence to keep a favorable context with larger thermodynamic advantages (see Figure 7). Thus, the stability variation introduced by GC was more dominant that that caused by the nucleotide order.

In order to check whether the regulation role of GC content in splicing is a universal phenomenon in multiple species, we extended our work on humans to nematodes, fruit files, and mice. The results of energy vs. GC were further summarized in a table (Additional file 11). In spite of the lower quality of the splicing event lists due to the incompleteness of the gene annotation compared to humans, we also observed the enrichment of stable structures in alternatively spliced sites as well as slightly different yet still statistically significant correlations between the GC percentage and the free energy in all these species. Thus, the involvement of GC content in the splicing regulation process might have been in existence from ancient times.

## Conclusions

All together, our results show that GC content around splice sites may play an important role in splicing regulation by forming stable secondary structures. Through the selection of GC enriched sequences, exons with alternative splice sites can maintain stable pre-mRNA structures to promote alternative splicing. This GC effect is more dominant than the nucleotide-order effect. On the other hand, constitutive exons and cassette exons are not enriched with stable structures. It indicates that that the pre-mRNA structure is part of, but not the whole of, the splicing code. We expect to investigate the biological significance in details when related experimental data become available in the future.

## Methods

### Splice site assembly

Splice site positions of different human exons were obtained from the UCSC Genome Browser (alternative splicing event track, version hg18). For alternative splice sites, we required a distance of at least 8 bp between two 3' splice sites of the same exon, and at least 5 bp between two 5' splice sites of the same exon. A total of 4,128 internal alternative 5'ss, 5,899 internal alternative 3'ss, and 44,337 skipped splice sites (21,473 5'ss and 22,864 3'ss) for cassette exons were extracted. We generated our own list of internal constitutive exons using the UCSC transcript annotation information with two requirements: (1) a constitutive exon should appear in all isoforms (with at least three exons) of the gene without overlapping with any other exon and preserve exactly the same starting and ending positions; (2) the gene should contain at least four different isoforms. Thus, 30,992 internal constitutive splicing sites (15,496 5' ss and 15,496 3'ss) were selected in total. Note that for exons with alternative splice sites, all of the splice sites were used for the structure and GC analysis.

For the donor sites of the first exons study, we generated the list of first alternative and constitutive sites by examining transcript isoforms. We focused on transcripts with at least three exons. Only the first exons which share the same transcription starting sites in all the transcript isoforms of the same genes were considered. Constitutive first donor sites were defined as the ones appearing at the 5'ss of the initial exons and keeping exactly the same position in all transcript isoforms (at least three isoforms). If the donor site of the first did not appear in all of the transcript isoforms, it was counted as an alternative first donor site. According to these criteria, 405 alternative first donor sites and 1,761 constitutive first donor sites were generated in our work.

Refseq gene annotations (version WS190) were downloaded from the UCSC genome browser http://genome.ucsc.edu/ in the analysis of nematodes. For the fruit fly and mouse studies, gene annotation lists were downloaded from http://genome.ewha.ac.kr/ECgene/, in which only transcripts with high quality annotations (confidence high group) were selected. Alternative splice sites and skipped splice sites were assembled from the annotation lists. Similar criteria as in humans were used to generate the constitutive exons lists.

### Pre-mRNA secondary structure prediction

NCBI build 36 for the mouse and human, BDGP Release 4 for the fruit fly, and WS190 for the nematode genome sequences were used for the structure prediction. For each splice site, 70 nucleotides were added both up and downstream of the intron-exon junction to form a 141 nt window, and then the RNAfold program was used to predict the minimum free energy. The default settings of RNAfold were used for the energy prediction in humans and mice. However, for nematodes and fruit flies, the prediction temperature was set to 25 and 24 degrees centigrade respectively to adjust for the different temperatures for growth.

### Tissue-specific alternative splice sites and decoy splice sites

Tissue-specific alternative spliced sites were obtained from the microarray study where the expression of alternative splicing events in 48 different human cells was profiled [24]. For each alternative donor or acceptor sites, if the isoform proportionality change was greater than 10 percent and at the same time the corresponding P value was less than 0.3, we claimed that they were tissue-specific alternative splice sites. The non-tissue specific alternative splice sites were those with the proportionality change less than 5 percent or the P value were larger than 0.3.

As a control group of real splice sites, decoy splice sites that share similar consensus sequence information but seldom experience the splicing events were selected. Firstly, all the AG/GU dinucleotides in the flanking introns within 300-150 nt to the real splice site were extracted as candidates for decoy splice sites, and MaxEntScan code from http://genes.mit.edu/burgelab/maxent/Xmaxentscan_scoreseq.html was used to compute the splice sites score for each candidate. A higher score indicates a larger similarity to the consensus sequence of their training data. The candidates with a higher score within 300-150 nt to the real splice sites were chosen as decoy splice sites. For the splice sites with multiple decoy sites nearby, only the nearest one was selected as the control to the real junction.

### Nucleotide-order effect

Two random permutations were used to generate the control data to evaluate the nucleotide-order effect on secondary structures. In the permutation that kept only the first order nucleotide frequencies, each 141 nt sequence was randomly permuted and the pre-mRNA secondary structure was predicted for the permuted sequence. We did the permutation ten times. The results were all similar (data not shown). It is well known that the dinucleotide frequency affects the predicted stability significantly by the RNAfold program [27]. Thus for a fair comparison, the ushuffle program at http://digital.cs.usu.edu/~mjiang/ushuffle/ was used to generate the permutations that fixed the dinucleotide frequencies.

### Statistical analyses

All of the statistical analyses including the Wilcoxon tests, the pairwise Wilcoxon tests, and regression analyses were performed using the R software.

## Abbreviations

GC content: guanine-cytosine content; 5ss: 5' splice site; 3ss: 3' splice site; nt: nucleotide; bp: base pair.

## Authors' contributions

LC and JZ designed the overall content of this paper. JZ performed the data analysis. LC and CJK supervised the study. JZ, LC, and CJK drafted the paper together. All authors read and approved the final manuscript.

## Supplementary Material

Additional file 1 **(Figure) Comparison of stability distribution of alternative splice sites and constitutive or skipped splice sites in nematodes at 25°C**. At the donor sites (5'ss), alternative splice sites exhibited more stable structures than constitutive and skipped sites (-42.18 vs. -40.21 and -39.66 kcals/mol, Wilcoxon test *P *values were 1.26 × 10^-7 ^and 3.51 × 10^-11 ^respectively). For the comparison between alt3 and cons3, the average energy was -40.06 vs. -38.46 kcals/mol, Wilcoxon test *P *< 2.2 × 10^-16^. For the comparison between alt3 and skip3, the average energy was -40.06 vs. -37.81 kcals/mol, Wilcoxon test *P *< 2.2 × 10^-16^. Click here for file

Additional file 2 **(Figure) Comparison of stability distribution of alternative splice sites and constitutive or skipped splice sites in fruit flies at 24°C**. Alternative splice sites exhibited more stable structures compared with constitutive and skipped splice sites. The average energy for the alternative, constitutive, and skipped donor sites was -49.74, -46.58, and -44.53 kcals/mol respectively. The Wilcoxon test *P*-value was 2.7 × 10^-10 ^and 2.2 × 10^-16^. For the comparison between alt3 and cons3, the average energy was -45.50 vs. -44.17 kcals/mol, Wilcoxon test *P *= 5.2 × 10^-4^. For the comparison between alt3 and skip3, the average energy was -45.50 vs. -41.65 kcals/mol, Wilcoxon test *P *< 2.2 × 10^-16^. Click here for file

Additional file 3 **(Figure) Comparison of stability distribution of alternative splice sites and constitutive or skipped splice sites in mice at 37°C**. At the acceptor sites (3'ss), alternative splice sites exhibited more stable structures than constitutive and skipped sites (-38.43 vs. -36.07 and -35.80 kcals/mol, Wilcoxon test *P *< 2.2 × 10^-16^). The average energy for the alternative and skipped donor sites was -39.10 and -37.74 kcals/mol respectively (Wilcoxon test *P *= 1.1 × 10^-11^). However, the difference between alternative and constitutive donor sites was small (-39.10 vs. -38.76 kcals/mol, Wilcoxon test, *P *= 0.20). Click here for file

Additional file 4 **(Figure) Distributions of the GC content of different splice sites in humans (window size = 61 nt)**. At the donor sites, the average GC content for alternative, constitutive and skipped sites was 0.52, 0.48, and 0.47 respectively. At the acceptor sites, the average GC content for alternative, constitutive and skipped sites was 0.51, 0.46, and 0.45 respectively. For both sites, the p-values of the Wilcoxon tests between alternative and constitutive or skipped sites were all less than 2.2 × 10^-16^. Click here for file

Additional file 5 **(Table) Comparison between alternative splice sites and constitutive or skipped splice sites in humans in terms of exonic GC, intronic GC, overall GC, as well as the correlation with the structural energy**. The p-values for the correlation test were all less than 2.2 × 10^-16^. Alternative splice sites had higher GC content compared with constitutive or skipped splice sites in exonic region, intronic region and the whole splice site window. But the overall GC in the whole window exhibited the highest absolute correlation with the structural stability. Click here for file

Additional file 6 **(Figure) Scatter plots of the energy and the GC content in nematodes at 25°C**. A-C are for alternative, constitutive, and skipped 5'ss. D-F are for alternative, constitutive, and skipped 3'ss. Click here for file

Additional file 7 **(Figure) Scatter plots of the energy and the GC content in fruit flies at 24°C**. A-C are for alternative, constitutive, and skipped 5'ss. D-F are for alternative, constitutive, and skipped 3'ss. Click here for file

Additional file 8 **(Figure) Scatter plots of the energy and the GC content in mice at 37°C**. A-C are for alternative, constitutive, and skipped 5'ss. D-F are for alternative, constitutive, and skipped 3'ss. Click here for file

Additional file 9 **(Figure) Scatter plots of the energy and the GC content in nematodes at 37°C**. A-C are for alternative, constitutive, and skipped 5'ss. D-F are for alternative, constitutive, and skipped 3'ss. Click here for file

Additional file 10 **(Figure) Scatter plots of the energy and the GC content in fruit flies at 37°C**. A-C are for alternative, constitutive, and skipped 5'ss. D-F are for alternative, constitutive, and skipped 3'ss. Click here for file

Additional file 11 **(Table) Summary of energy analysis results in humans, mice, fruit flies, and nematodes**. Click here for file

## References

[B1] Jurica MS Moore MJ Pre-mRNA splicing: awash in a sea of proteins Mol Cell 2003 12 1 5 14 10.1016/S1097-2765(03)00270-3 12887888

[B2] Zhou Z Licklider LJ Gygi SP Reed R Comprehensive proteomic analysis of the human spliceosome Nature 2002 419 6903 182 185 10.1038/nature01031 12226669

[B3] Cartegni L Chew SL Krainer AR Listening to silence and understanding nonsense: exonic mutations that affect splicing Nat Rev Genet 2002 3 4 285 298 10.1038/nrg775 11967553

[B4] Faustino NA Cooper TA Pre-mRNA splicing and human disease Genes Dev 2003 17 4 419 437 10.1101/gad.1048803 12600935

[B5] Stadler MB Shomron N Yeo GW Schneider A Xiao X Burge CB Inference of splicing regulatory activities by sequence neighborhood analysis PLoS Genet 2006 2 11 e191 10.1371/journal.pgen.0020191 17121466 PMC1657047

[B6] Wang Z Burge CB Splicing regulation: from a parts list of regulatory elements to an integrated splicing code RNA 2008 14 5 802 813 10.1261/rna.876308 18369186 PMC2327353

[B7] Buratti E Baralle FE Influence of RNA secondary structure on the pre-mRNA splicing process Mol Cell Biol 2004 24 24 10505 10514 10.1128/MCB.24.24.10505-10514.2004 15572659 PMC533984

[B8] Clouet d'Orval B d'Aubenton Carafa Y Sirand-Pugnet P Gallego M Brody E Marie J RNA secondary structure repression of a muscle-specific exon in HeLa cell nuclear extracts Science 1991 252 5014 1823 1828 2063195 10.1126/science.2063195

[B9] Eperon LP Graham IR Griffiths AD Eperon IC Effects of RNA secondary structure on alternative splicing of pre-mRNA: is folding limited to a region behind the transcribing RNA polymerase? Cell 1988 54 3 393 401 10.1016/0092-8674(88)90202-4 2840206

[B10] Graveley BR Mutually exclusive splicing of the insect Dscam pre-mRNA directed by competing intronic RNA secondary structures Cell 2005 123 1 65 73 10.1016/j.cell.2005.07.028 16213213 PMC2366815

[B11] Jacquenet S Ropers D Bilodeau PS Damier L Mougin A Stoltzfus CM Branlant C Conserved stem-loop structures in the HIV-1 RNA region containing the A3 3' splice site and its cis-regulatory element: possible involvement in RNA splicing Nucleic Acids Res 2001 29 2 464 478 10.1093/nar/29.2.464 11139617 PMC29680

[B12] Loeb DD Mack AA Tian R A secondary structure that contains the 5' and 3' splice sites suppresses splicing of duck hepatitis B virus pregenomic RNA J Virol 2002 76 20 10195 10202 10.1128/JVI.76.20.10195-10202.2002 12239294 PMC136586

[B13] Patterson DJ Yasuhara K Ruzzo WL Pre-mRNA secondary structure prediction aids splice site prediction Pac Symp Biocomput 2002 223 234 11928478

[B14] Hiller M Zhang Z Backofen R Stamm S Pre-mRNA secondary structures influence exon recognition PLoS Genet 2007 3 11 e204 10.1371/journal.pgen.0030204 18020710 PMC2077896

[B15] Raker VA Mironov AA Gelfand MS Pervouchine DD Modulation of alternative splicing by long-range RNA structures in Drosophila Nucleic Acids Res 2009 37 14 4533 4544 10.1093/nar/gkp407 19465384 PMC2724269

[B16] Shepard PJ Hertel KJ Conserved RNA secondary structures promote alternative splicing RNA 2008 14 8 1463 1469 10.1261/rna.1069408 18579871 PMC2491482

[B17] Warf MB Berglund JA Role of RNA structure in regulating pre-mRNA splicing Trends Biochem Sci 2009 35 3 169 178 10.1016/j.tibs.2009.10.004 19959365 PMC2834840

[B18] Barash Y Calarco JA Gao W Pan Q Wang X Shai O Blencowe BJ Frey BJ Deciphering the splicing code Nature 2010 465 7294 53 59 10.1038/nature09000 20445623

[B19] Kalari KR Casavant M Bair TB Keen HL Comeron JM Casavant TL Scheetz TE First exons and introns--a survey of GC content and gene structure in the human genome In Silico Biol 2006 6 3 237 242 16922687

[B20] Schroeder R Grossberger R Pichler A Waldsich C RNA folding in vivo Curr Opin Struct Biol 2002 12 3 296 300 10.1016/S0959-440X(02)00325-1 12127447

[B21] Bompfunewerer AF Backofen R Bernhart SH Hertel J Hofacker IL Stadler PF Will S Variations on RNA folding and alignment: lessons from Benasque J Math Biol 2008 56 1-2 129 144 10.1007/s00285-007-0107-5 17611759

[B22] Hofacker IL Stadler PF Memory efficient folding algorithms for circular RNA secondary structures Bioinformatics 2006 22 10 1172 1176 10.1093/bioinformatics/btl023 16452114

[B23] Rach EA Yuan HY Majoros WH Tomancak P Ohler U Motif composition, conservation and condition-specificity of single and alternative transcription start sites in the Drosophila genome Genome Biol 2009 10 7 R73 10.1186/gb-2009-10-7-r73 19589141 PMC2728527

[B24] Castle JC Zhang C Shah JK Kulkarni AV Kalsotra A Cooper TA Johnson JM Expression of 24,426 human alternative splicing events and predicted cis regulation in 48 tissues and cell lines Nat Genet 2008 40 12 1416 1425 10.1038/ng.264 18978788 PMC3197713

[B25] Yeo G Burge CB Maximum entropy modeling of short sequence motifs with applications to RNA splicing signals J Comput Biol 2004 11 2-3 377 394 10.1089/1066527041410418 15285897

[B26] Seffens W Digby D mRNAs have greater negative folding free energies than shuffled or codon choice randomized sequences Nucleic Acids Res 1999 27 7 1578 1584 10.1093/nar/27.7.1578 10075987 PMC148359

[B27] Workman C Krogh A No evidence that mRNAs have lower folding free energies than random sequences with the same dinucleotide distribution Nucleic Acids Res 1999 27 24 4816 4822 10.1093/nar/27.24.4816 10572183 PMC148783

[B28] Kertesz M Wan Y Mazor E Rinn JL Nutter RC Chang HY Segal E Genome-wide measurement of RNA secondary structure in yeast Nature 2010 467 7311 103 107 10.1038/nature09322 20811459 PMC3847670

[B29] Li X Quon G Lipshitz HD Morris Q Predicting in vivo binding sites of RNA-binding proteins using mRNA secondary structure RNA 2010 16 6 1096 1107 10.1261/rna.2017210 20418358 PMC2874161

[B30] Thompson-Jager S Domdey H Yeast pre-mRNA splicing requires a minimum distance between the 5' splice site and the internal branch acceptor site Mol Cell Biol 1987 7 11 4010 4016 3323885 10.1128/mcb.7.11.4010-4016.1987 PMC368070

[B31] Rogic S Montpetit B Hoos HH Mackworth AK Ouellette BF Hieter P Correlation between the secondary structure of pre-mRNA introns and the efficiency of splicing in Saccharomyces cerevisiae BMC Genomics 2008 9 355 10.1186/1471-2164-9-355 18664289 PMC2536676

[B32] Furger A O'Sullivan JM Binnie A Lee BA Proudfoot NJ Promoter proximal splice sites enhance transcription Genes Dev 2002 16 21 2792 2799 10.1101/gad.983602 12414732 PMC187476

[B33] Klopper AV Bois JS Grill SW Influence of secondary structure on recovery from pauses during early stages of RNA transcription Phys Rev E Stat Nonlin Soft Matter Phys 2010 81 3 Pt 1 030904 10.1103/PhysRevE.81.030904 20365690

[B34] Mahen EM Watson PY Cottrell JW Fedor MJ mRNA secondary structures fold sequentially but exchange rapidly in vivo PLoS Biol 2010 8 2 e1000307 10.1371/journal.pbio.1000307 20161716 PMC2817708

