# Supplementary material for: GC content around splice sites affects splicing through pre-mRNA secondary structures
Source: BMC Genomics. 2011 Jan 31;12:90. doi: 10.1186/1471-2164-12-90 (PMC3041747; doi:10.1186/1471-2164-12-90)
Supplement: Additional file 1 — (Figure) Comparison of stability distribution of alternative splice sites and constitutive or skipped splice sites in nematodes at 25°C. At the donor sites (5'ss), alternative splice sites exhibited more stable structures than constitutive and skipped sites (-42.18 vs. -40.21 and -39.66 kcals/mol, Wilcoxon test P values were 1.26 × 10^-7^ and 3.51 × 10^-11^ respectively). For the comparison between alt3 and cons3, the average energy was -40.06 vs. -38.46 kcals/mol, Wilcoxon test P < 2.2 × 10^-16^. For the comparison between alt3 and skip3, the average energy was -40.06 vs. -37.81 kcals/mol, Wilcoxon test P < 2.2 × 10^-16^. [file 1471-2164-12-90-S1.PPT]

## Slide 1
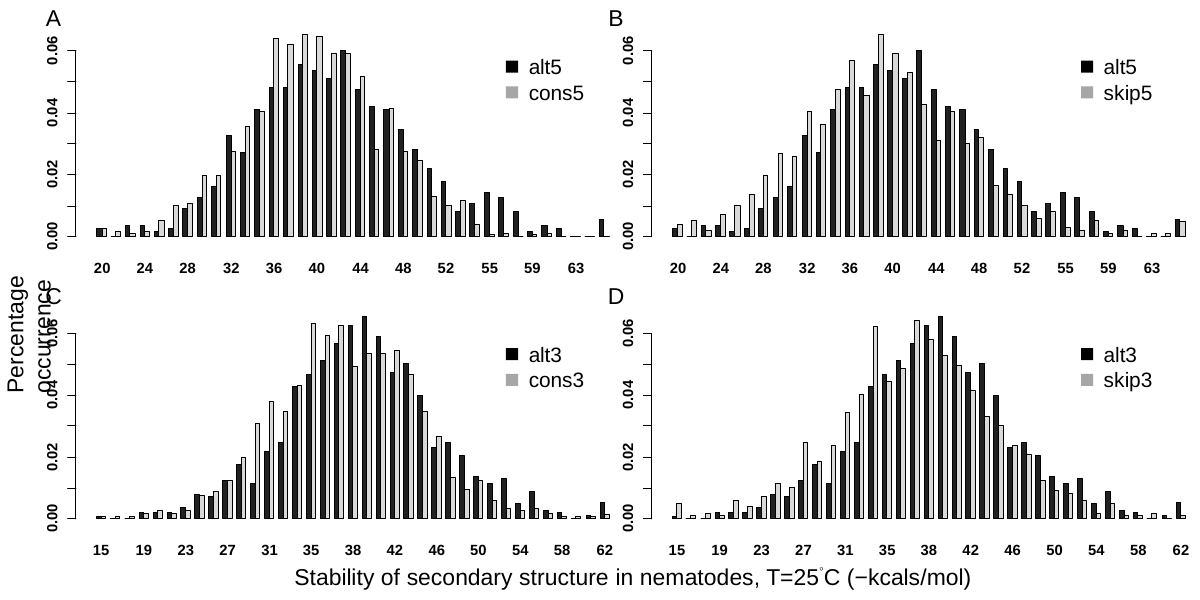

A
B
 alt5
 cons5
 alt5
 skip5
Percentage occurrence
C
D
 alt3
 cons3
 alt3
 skip3
Stability of secondary structure in nematodes, T=25◦C (−kcals/mol)
