# Supplementary material for: GC content around splice sites affects splicing through pre-mRNA secondary structures
Source: BMC Genomics. 2011 Jan 31;12:90. doi: 10.1186/1471-2164-12-90 (PMC3041747; doi:10.1186/1471-2164-12-90)
Supplement: Additional file 2 — (Figure) Comparison of stability distribution of alternative splice sites and constitutive or skipped splice sites in fruit flies at 24°C. Alternative splice sites exhibited more stable structures compared with constitutive and skipped splice sites. The average energy for the alternative, constitutive, and skipped donor sites was -49.74, -46.58, and -44.53 kcals/mol respectively. The Wilcoxon test P-value was 2.7 × 10^-10^ and 2.2 × 10^-16^. For the comparison between alt3 and cons3, the average energy was -45.50 vs. -44.17 kcals/mol, Wilcoxon test P = 5.2 × 10^-4^. For the comparison between alt3 and skip3, the average energy was -45.50 vs. -41.65 kcals/mol, Wilcoxon test P < 2.2 × 10^-16^. [file 1471-2164-12-90-S2.PPT]

## Slide 1
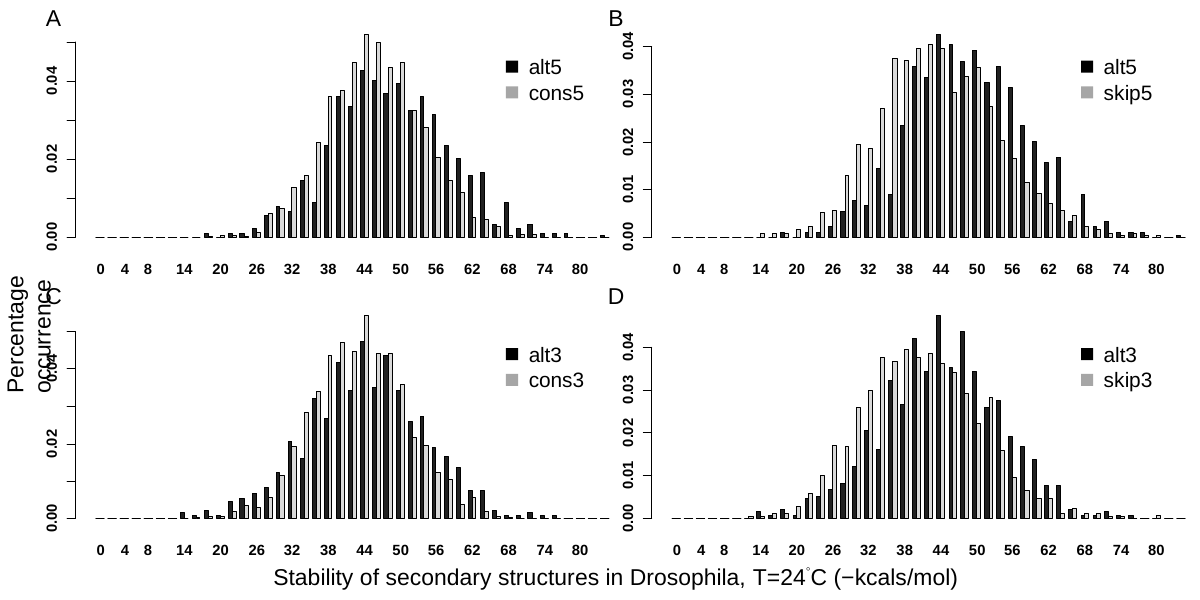

A
B
 alt5
 cons5
 alt5
 skip5
Percentage occurrence
C
D
 alt3
 cons3
 alt3
 skip3
Stability of secondary structures in Drosophila, T=24◦C (−kcals/mol)
