# Supplementary material for: GC content around splice sites affects splicing through pre-mRNA secondary structures
Source: BMC Genomics. 2011 Jan 31;12:90. doi: 10.1186/1471-2164-12-90 (PMC3041747; doi:10.1186/1471-2164-12-90)
Supplement: Additional file 3 — (Figure) Comparison of stability distribution of alternative splice sites and constitutive or skipped splice sites in mice at 37°C. At the acceptor sites (3'ss), alternative splice sites exhibited more stable structures than constitutive and skipped sites (-38.43 vs. -36.07 and -35.80 kcals/mol, Wilcoxon test P < 2.2 × 10^-16^). The average energy for the alternative and skipped donor sites was -39.10 and -37.74 kcals/mol respectively (Wilcoxon test P = 1.1 × 10^-11^). However, the difference between alternative and constitutive donor sites was small (-39.10 vs. -38.76 kcals/mol, Wilcoxon test, P = 0.20). [file 1471-2164-12-90-S3.PPT]

## Slide 1
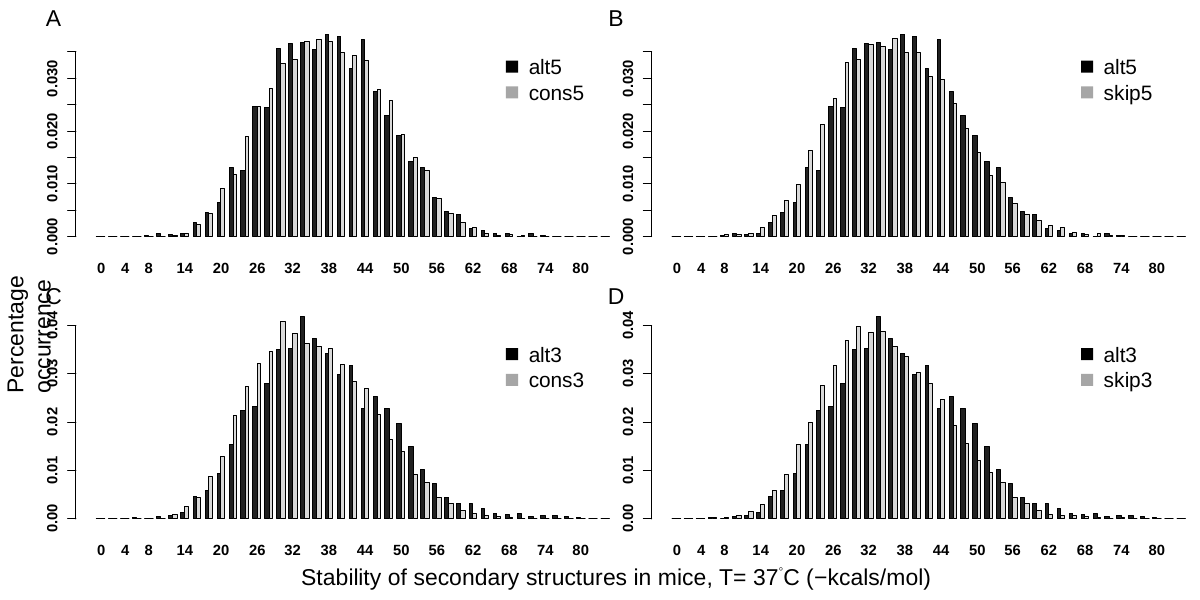

A
B
 alt5
 cons5
 alt5
 skip5
Percentage occurrence
C
D
 alt3
 cons3
 alt3
 skip3
Stability of secondary structures in mice, T= 37◦C (−kcals/mol)
