# Supplementary material for: GC content around splice sites affects splicing through pre-mRNA secondary structures
Source: BMC Genomics. 2011 Jan 31;12:90. doi: 10.1186/1471-2164-12-90 (PMC3041747; doi:10.1186/1471-2164-12-90)
Supplement: Additional file 4 — (Figure) Distributions of the GC content of different splice sites in humans (window size = 61 nt). At the donor sites, the average GC content for alternative, constitutive and skipped sites was 0.52, 0.48, and 0.47 respectively. At the acceptor sites, the average GC content for alternative, constitutive and skipped sites was 0.51, 0.46, and 0.45 respectively. For both sites, the p-values of the Wilcoxon tests between alternative and constitutive or skipped sites were all less than 2.2 × 10^-16^. [file 1471-2164-12-90-S4.PPT]

## Slide 1
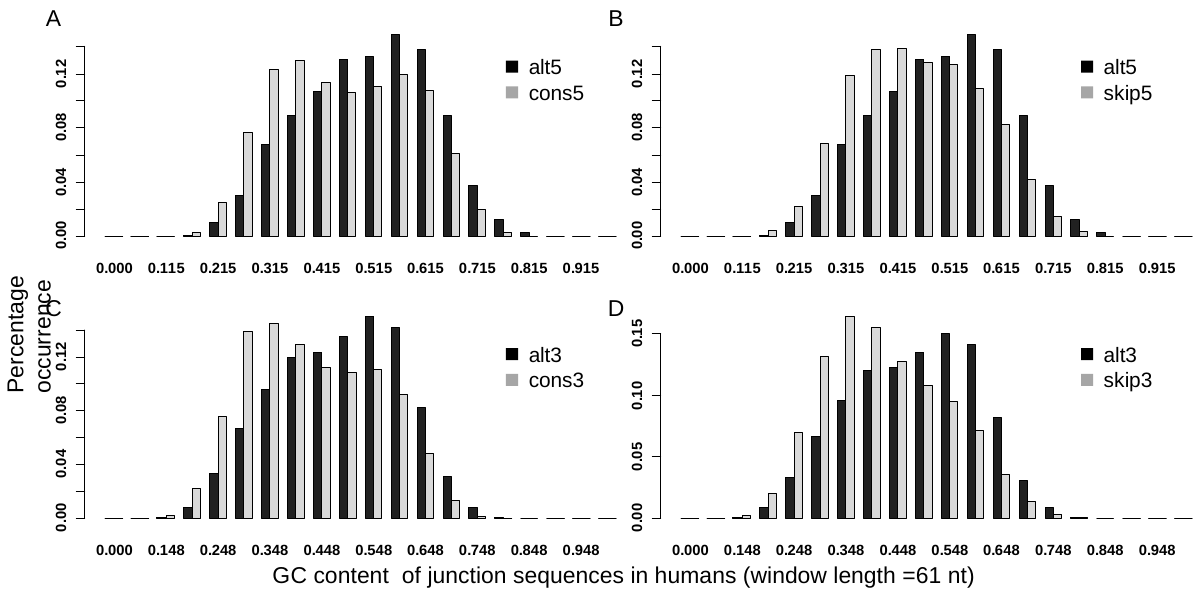

A
B
 alt5
 cons5
 alt5
 skip5
Percentage occurrence
C
D
 alt3
 cons3
 alt3
 skip3
GC content of junction sequences in humans (window length =61 nt)
