# Supplementary material for: GC content around splice sites affects splicing through pre-mRNA secondary structures
Source: BMC Genomics. 2011 Jan 31;12:90. doi: 10.1186/1471-2164-12-90 (PMC3041747; doi:10.1186/1471-2164-12-90)
Supplement: Additional file 5 — (Table) Comparison between alternative splice sites and constitutive or skipped splice sites in humans in terms of exonic GC, intronic GC, overall GC, as well as the correlation with the structural energy. The p-values for the correlation test were all less than 2.2 × 10^-16^. Alternative splice sites had higher GC content compared with constitutive or skipped splice sites in exonic region, intronic region and the whole splice site window. But the overall GC in the whole window exhibited the highest absolute correlation with the structural stability. [file 1471-2164-12-90-S5.DOC]

|  | | Alternative splice sites | | Constitutive splice sites | | Skipped splice sites | |
| --- | --- | --- | --- | --- | --- | --- | --- |
|  |  | Mean value | Correlation with energy | Mean value | Correlation with energy | Mean value | Correlation with energy |
|  | Exonic GC | 0.53 | −0.81 | 0.50 | −0.81 | 0.48 | −0.78 |
| 5’ ss | Intronic GC | 0.51 | −0.84 | 0.46 | −0.86 | 0.45 | −0.83 |
|  | Overall GC | 0.52 | −0.87 | 0.48 | −0.90 | 0.47 | −0.88 |
|  | Exonic GC | 0.54 | −0.75 | 0.50 | −0.79 | 0.49 | −0.76 |
| 3’ss | Intronic GC | 0.50 | −0.81 | 0.44 | −0.84 | 0.42 | −0.80 |
|  | Overall GC | 0.52 | −0.83 | 0.47 | −0.88 | 0.46 | −0.85 |
