# Supplementary material for: GC content around splice sites affects splicing through pre-mRNA secondary structures
Source: BMC Genomics. 2011 Jan 31;12:90. doi: 10.1186/1471-2164-12-90 (PMC3041747; doi:10.1186/1471-2164-12-90)
Supplement: Additional file 6 — (Figure) Scatter plots of the energy and the GC content in nematodes at 25°C. A-C are for alternative, constitutive, and skipped 5'ss. D-F are for alternative, constitutive, and skipped 3'ss. [file 1471-2164-12-90-S6.PPT]

## Slide 1
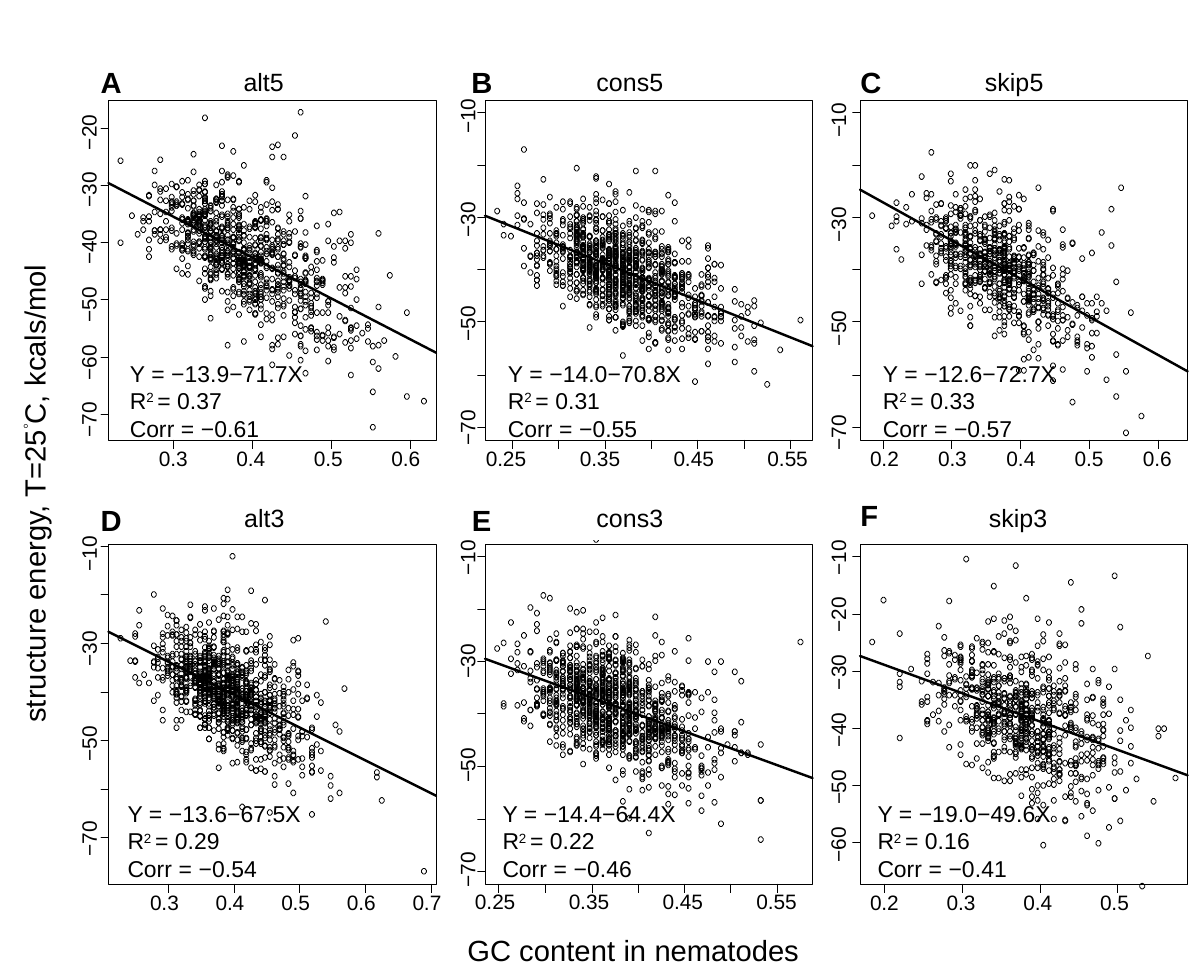

A
B
C
alt5
cons5
skip5
−10
−30
−50
−70
−10
−30
−50
−70
−20
−30
−40
−50
−60
−70
structure energy, T=25◦C, kcals/mol
Y = −13.9−71.7X
R2 = 0.37
Corr = −0.61
Y = −14.0−70.8X
R2 = 0.31
Corr = −0.55
Y = −12.6−72.7X
R2 = 0.33
Corr = −0.57
0.3
0.4
0.5
0.6
0.25
0.35
0.45
0.55
0.2
0.3
0.4
0.5
0.6
F
D
alt3
E
cons3
skip3
−10
−30
−50
−70
−10
−20
−30
−40
−50
−60
−10
−30
−50
−70
Y = −13.6−67.5X
R2 = 0.29
Corr = −0.54
Y = −14.4−64.4X
R2 = 0.22
Corr = −0.46
Y = −19.0−49.6X
R2 = 0.16
Corr = −0.41
0.25
0.35
0.45
0.55
0.3
0.4
0.5
0.6
0.7
0.2
0.3
0.4
0.5
GC content in nematodes
