# Supplementary material for: GC content around splice sites affects splicing through pre-mRNA secondary structures
Source: BMC Genomics. 2011 Jan 31;12:90. doi: 10.1186/1471-2164-12-90 (PMC3041747; doi:10.1186/1471-2164-12-90)
Supplement: Additional file 7 — (Figure) Scatter plots of the energy and the GC content in fruit flies at 24°C. A-C are for alternative, constitutive, and skipped 5'ss. D-F are for alternative, constitutive, and skipped 3'ss. [file 1471-2164-12-90-S7.PPT]

## Slide 1
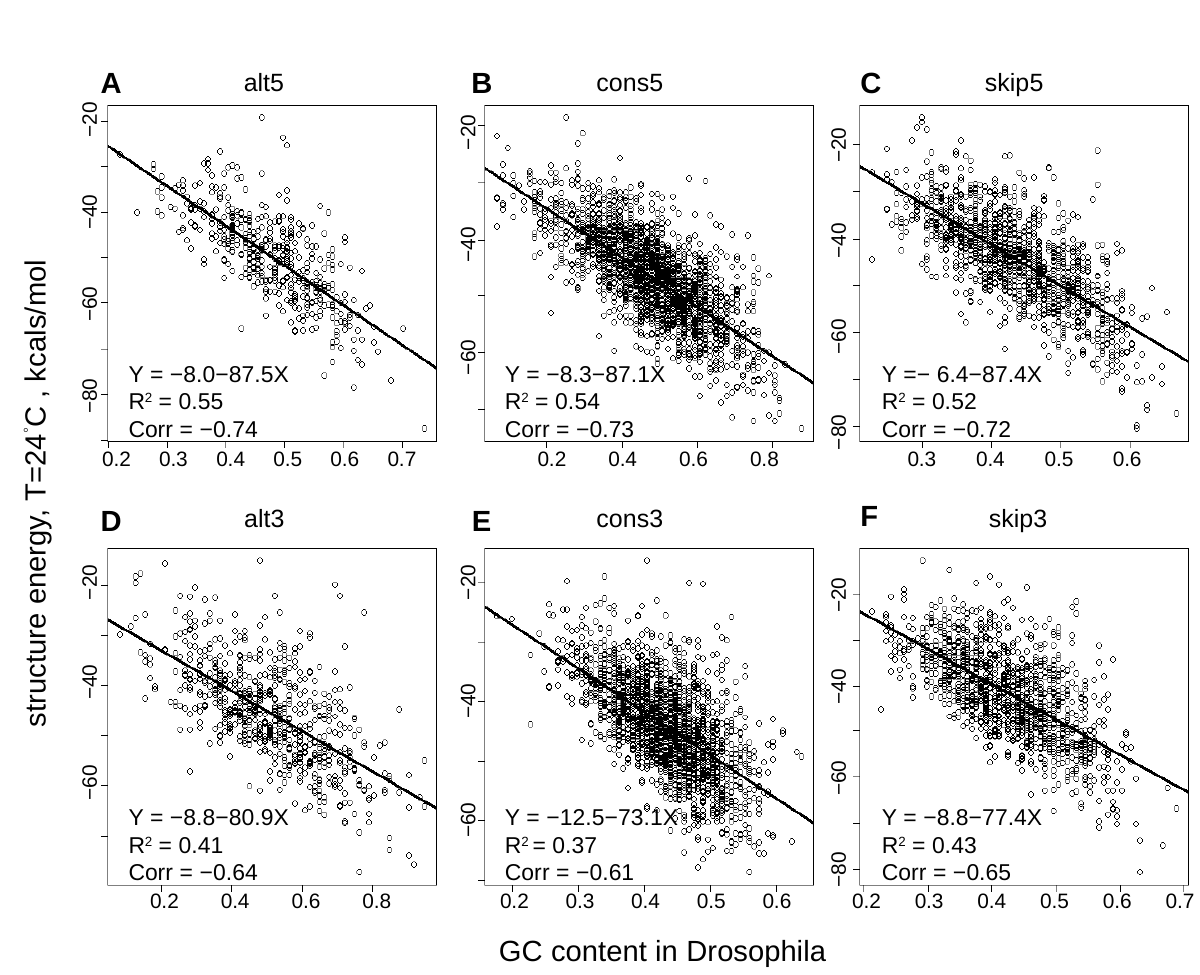

A
B
C
alt5
cons5
skip5
−20
−20
−20
structure energy, T=24◦C , kcals/mol
−40
−40
−40
−60
−60
−60
Y = −8.0−87.5X
R2 = 0.55
Corr = −0.74
Y = −8.3−87.1X
R2 = 0.54
Corr = −0.73
Y =− 6.4−87.4X
R2 = 0.52
Corr = −0.72
−80
−80
0.2
0.3
0.4
0.5
0.6
0.7
0.2
0.4
0.6
0.8
0.3
0.4
0.5
0.6
F
D
alt3
E
cons3
skip3
−20
−20
−20
−40
−60
−80
−40
−40
−60
Y = −8.8−80.9X
R2 = 0.41
Corr = −0.64
Y = −12.5−73.1X
R2 = 0.37
Corr = −0.61
Y = −8.8−77.4X
R2 = 0.43
Corr = −0.65
−60
0.2
0.4
0.6
0.8
0.2
0.3
0.4
0.5
0.6
0.2
0.3
0.4
0.5
0.6
0.7
GC content in Drosophila
