# Supplementary material for: GC content around splice sites affects splicing through pre-mRNA secondary structures
Source: BMC Genomics. 2011 Jan 31;12:90. doi: 10.1186/1471-2164-12-90 (PMC3041747; doi:10.1186/1471-2164-12-90)
Supplement: Additional file 8 — (Figure) Scatter plots of the energy and the GC content in mice at 37°C. A-C are for alternative, constitutive, and skipped 5'ss. D-F are for alternative, constitutive, and skipped 3'ss. [file 1471-2164-12-90-S8.PPT]

## Slide 1
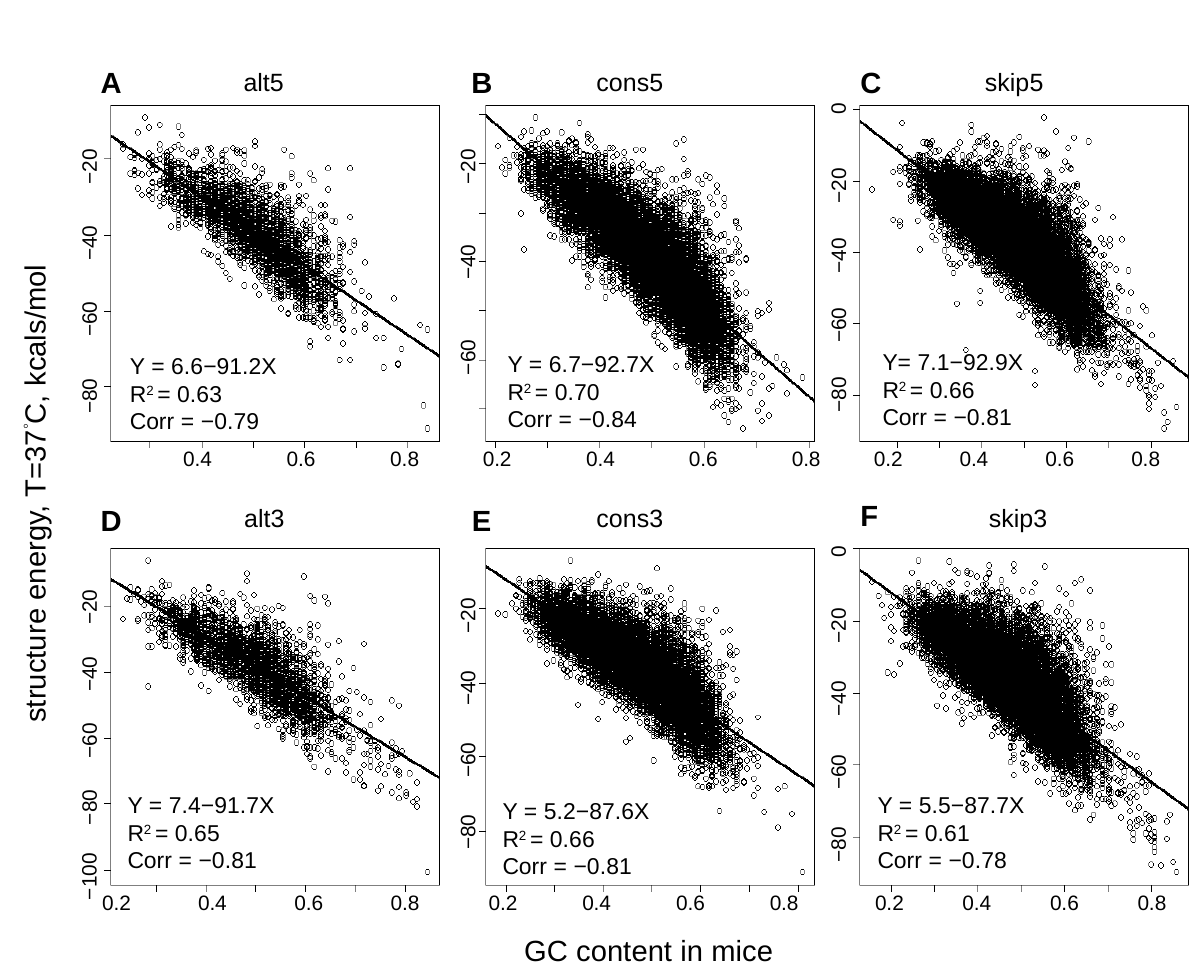

A
B
C
alt5
cons5
skip5
0
−20
−40
−60
−80
−20
−40
−60
−20
−40
structure energy, T=37◦C, kcals/mol
−60
Y= 7.1−92.9X
R2 = 0.66
Corr = −0.81
Y = 6.7−92.7X
R2 = 0.70
Corr = −0.84
Y = 6.6−91.2X
R2 = 0.63
Corr = −0.79
−80
0.4
0.6
0.8
0.2
0.4
0.6
0.8
0.2
0.4
0.6
0.8
F
D
alt3
E
cons3
skip3
0
−20
−40
−60
−80
−20
−20
−40
−60
−80
−40
−60
Y = 7.4−91.7X
R2 = 0.65
Corr = −0.81
Y = 5.5−87.7X
R2 = 0.61
Corr = −0.78
Y = 5.2−87.6X
R2 = 0.66
Corr = −0.81
−80
−100
0.2
0.4
0.6
0.8
0.2
0.4
0.6
0.8
0.2
0.4
0.6
0.8
GC content in mice
