# Supplementary material for: GC content around splice sites affects splicing through pre-mRNA secondary structures
Source: BMC Genomics. 2011 Jan 31;12:90. doi: 10.1186/1471-2164-12-90 (PMC3041747; doi:10.1186/1471-2164-12-90)
Supplement: Additional file 9 — (Figure) Scatter plots of the energy and the GC content in nematodes at 37°C. A-C are for alternative, constitutive, and skipped 5'ss. D-F are for alternative, constitutive, and skipped 3'ss. [file 1471-2164-12-90-S9.PPT]

## Slide 1
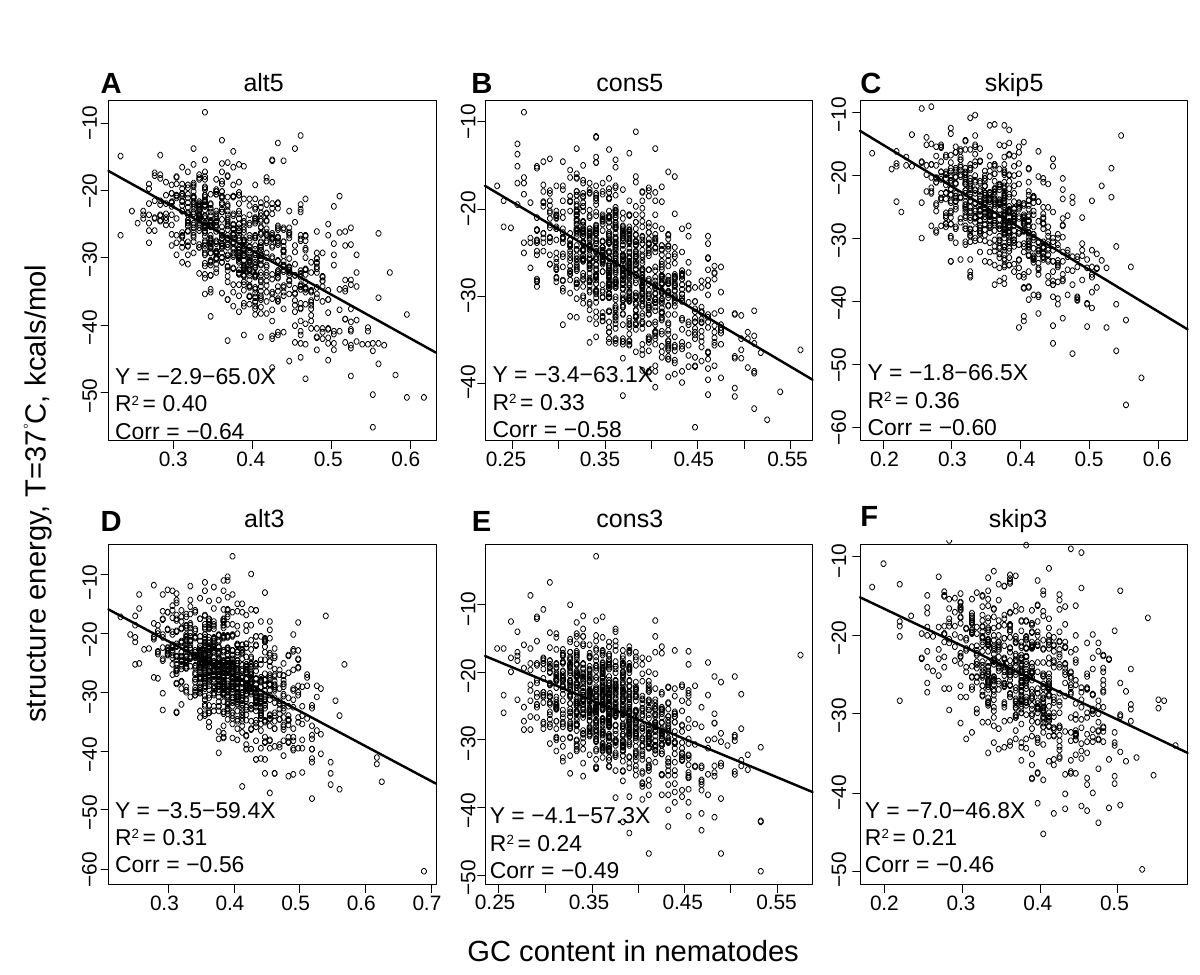

A
B
C
alt5
cons5
skip5
−10
−20
−30
−40
−50
−60
−10
−20
−30
−40
−10
−20
−30
−40
−50
structure energy, T=37◦C, kcals/mol
Y = −1.8−66.5X
R2 = 0.36
Corr = −0.60
Y = −3.4−63.1X
R2 = 0.33
Corr = −0.58
Y = −2.9−65.0X
R2 = 0.40
Corr = −0.64
0.3
0.4
0.5
0.6
0.25
0.35
0.45
0.55
0.2
0.3
0.4
0.5
0.6
F
D
alt3
E
cons3
skip3
−10
−20
−30
−40
−50
−10
−20
−30
−40
−50
−60
−10
−20
−30
−40
−50
Y = −3.5−59.4X
R2 = 0.31
Corr = −0.56
Y = −7.0−46.8X
R2 = 0.21
Corr = −0.46
Y = −4.1−57.3X
R2 = 0.24
Corr = −0.49
0.25
0.35
0.45
0.55
0.3
0.4
0.5
0.6
0.7
0.2
0.3
0.4
0.5
GC content in nematodes
