# Supplementary material for: GC content around splice sites affects splicing through pre-mRNA secondary structures
Source: BMC Genomics. 2011 Jan 31;12:90. doi: 10.1186/1471-2164-12-90 (PMC3041747; doi:10.1186/1471-2164-12-90)
Supplement: Additional file 10 — (Figure) Scatter plots of the energy and the GC content in fruit flies at 37°C. A-C are for alternative, constitutive, and skipped 5'ss. D-F are for alternative, constitutive, and skipped 3'ss. [file 1471-2164-12-90-S10.PPT]

## Slide 1
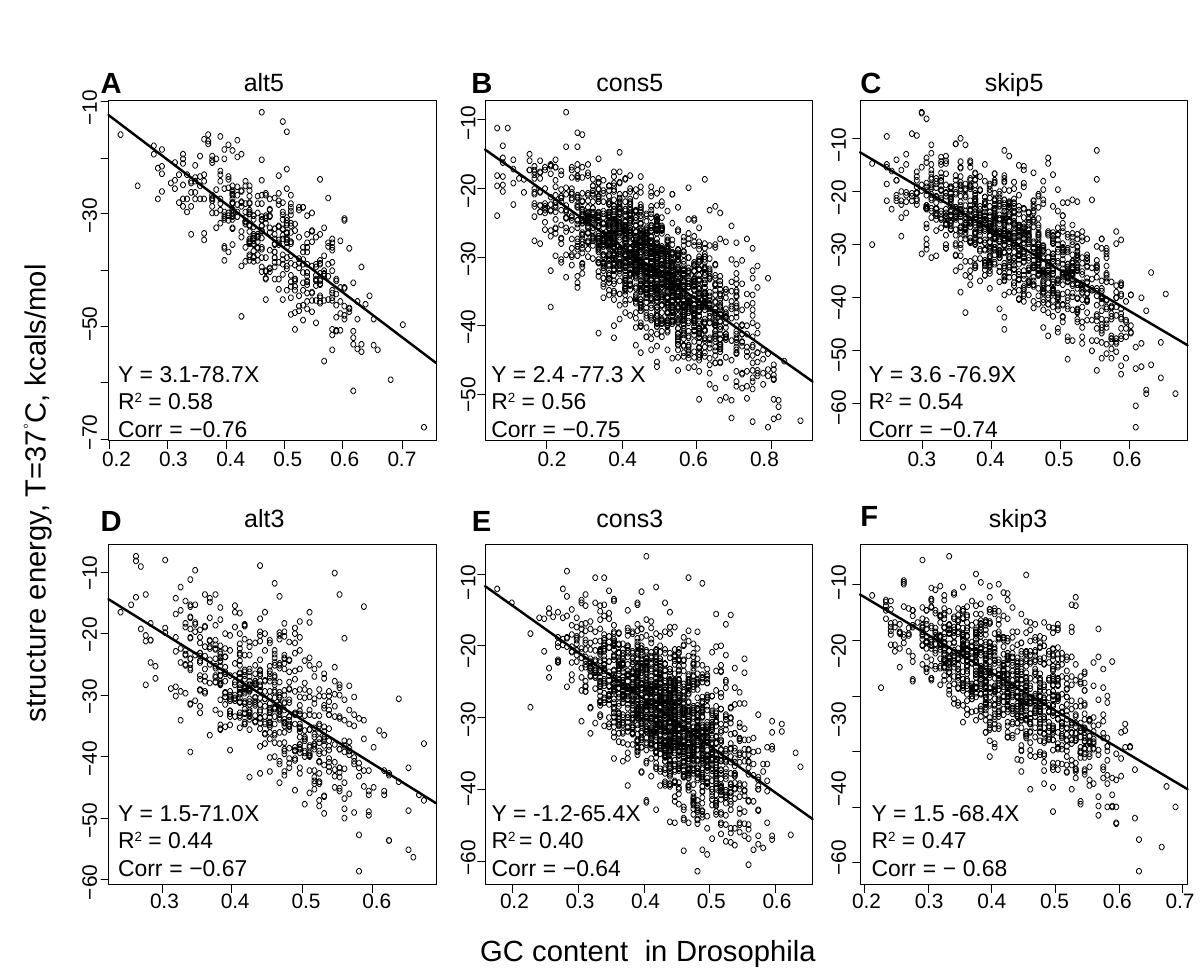

A
B
C
alt5
cons5
skip5
−10
−30
−50
−70
−10
−20
−30
−40
−50
−10
−20
−30
−40
−50
−60
structure energy, T=37◦C, kcals/mol
Y = 3.1-78.7X
R2 = 0.58
Corr = −0.76
Y = 2.4 -77.3 X
R2 = 0.56
Corr = −0.75
Y = 3.6 -76.9X
R2 = 0.54
Corr = −0.74
0.2
0.3
0.4
0.5
0.6
0.7
0.2
0.4
0.6
0.8
0.3
0.4
0.5
0.6
F
D
alt3
E
cons3
skip3
−10
−20
−30
−40
−50
−60
−10
−20
−30
−40
−60
−10
−20
−30
−40
−60
Y = 1.5-71.0X
R2 = 0.44
Corr = −0.67
Y = -1.2-65.4X
R2 = 0.40
Corr = −0.64
Y = 1.5 -68.4X
R2 = 0.47
Corr = − 0.68
0.3
0.4
0.5
0.6
0.2
0.3
0.4
0.5
0.6
0.2
0.3
0.4
0.5
0.6
0.7
GC content in Drosophila
