# Supplementary material for: GC content around splice sites affects splicing through pre-mRNA secondary structures
Source: BMC Genomics. 2011 Jan 31;12:90. doi: 10.1186/1471-2164-12-90 (PMC3041747; doi:10.1186/1471-2164-12-90)
Supplement: Additional file 11 — (Table) Summary of energy analysis results in humans, mice, fruit flies, and nematodes. [file 1471-2164-12-90-S11.DOC]

|  | | energy mean  (−kcals/mol) | | energy std | | GC mean | | GC std | | correlation | |
| --- | --- | --- | --- | --- | --- | --- | --- | --- | --- | --- | --- |
|  |  | 5’ss | 3’ss | 5’ss | 3’ss | 5’ss | 3’ss | 5’ss | 3’ss | 5’ss | 3’ss |
| humans | alt | 41.28 | 40.03 | 12.36 | 12.59 | 0.52 | 0.52 | 0.11 | 0.11 | 0.87 | 0.83 |
|  | cons | 38.43 | 36.18 | 12.74 | 12.24 | 0.48 | 0.47 | 0.12 | 0.12 | 0.90 | 0.88 |
|  | skip | 37.20 | 35.28 | 12.21 | 11.89 | 0.47 | 0.46 | 0.11 | 0.11 | 0.88 | 0.85 |
| mice | alt | 39.10 | 38.43 | 9.89 | 10.85 | 0.50 | 0.50 | 0.09 | 0.10 | 0.79 | 0.81 |
|  | cons | 38.76 | 36.07 | 9.67 | 9.73 | 0.49 | 0.47 | 0.09 | 0.09 | 0.84 | 0.81 |
|  | skip | 37.74 | 35.80 | 10.27 | 10.10 | 0.48 | 0.47 | 0.09 | 0.09 | 0.81 | 0.78 |
| fruit flies | alt | 49.74 | 45.50 | 9.92 | 9.97 | 0.48 | 0.45 | 0.08 | 0.08 | 0.74 | 0.64 |
|  | cons | 46.58 | 44.17 | 8.03 | 7.88 | 0.44 | 0.43 | 0.07 | 0.07 | 0.73 | 0.61 |
|  | skip | 44.53 | 41.65 | 9.99 | 9.75 | 0.44 | 0.42 | 0.08 | 0.08 | 0.72 | 0.65 |
| nematodes | alt | 42.18 | 40.06 | 7.60 | 7.31 | 0.39 | 0.39 | 0.06 | 0.06 | 0.61 | 0.54 |
|  | cons | 40.21 | 38.46 | 6.36 | 6.76 | 0.37 | 0.37 | 0.05 | 0.05 | 0.55 | 0.46 |
|  | skip | 39.66 | 37.81 | 7.97 | 7.50 | 0.37 | 0.38 | 0.06 | 0.06 | 0.57 | 0.41 |
